# Targeted delivery of liposomal senolytics to alleviate cellular senescence-induced bone loss

**DOI:** 10.1016/j.fmre.2024.12.010

**Published:** 2024-12-27

**Authors:** Rong Li, Yaohua Wei, Changhao Xiong, Jingwei Wang, Yixuan Lin, Ronghui Deng, Hao Qin, Yang Chen, Nan Li, Guyu Zheng, Yuanyuan Lv, Jian Shi, Tingting Yu, Yiye Li, Jing Wang, Ruifang Zhao, Changsheng Liu, Guangjun Nie

**Affiliations:** a College of Chemistry, Zhengzhou University, Zhengzhou 450001, China; b CAS Key Laboratory for Biomedical Effects of Nanomaterials and Nanosafety, CAS Center for Excellence in Nanoscience, National Center for Nanoscience and Technology, Beijing 100190, China; c Center of Materials Science and Optoelectronics Engineering, University of Chinese Academy of Sciences, Beijing 100049, China; d Department of Orthodontics, Peking University School and Hospital of Stomatology, Beijing 100081, China; e Key Laboratory for Ultrafine Materials of Ministry of Education, East China University of Science and Technology, Shanghai 200237, China

**Keywords:** Bone targeting, Cellular senescence, Dasatinib, Quercetin, Osteoporosis

## Abstract

The senescence of bone marrow-derived mesenchymal stem cells is involved in osteoporosis. The combination of dasatinib and quercetin has been explored to alleviate bone loss by efficiently reducing senescent cell populations. However, senolytic therapy by dasatinib and quercetin requires a precise ratio for better therapeutic effects, which is hard to achieve by oral administration. Meanwhile, the poor water solubility of these compounds limits their bioavailability, and their non-specific action could hamper effective penetration and targeting within relevant tissues. Herein, we developed alendronate-functionalized liposomes carrying dasatinib and quercetin (Aln-Lipo-DQ), focusing mainly on senescence-associated osteoporosis induced by chemotherapy or radiotherapy. Alendronate helps liposomes deliver dasatinib and quercetin to the femur and tibias, effectively removing senescent cells from bone tissue and increasing bone volume fraction from 5.05% to 11.95% in the chemotherapy-induced osteoporosis mouse model. We also found a 2.91-fold increase in bone volume fraction in Aln-Lipo-DQ treated groups compared to the control in radiotherapy models. This selectively targeting bone and reducing senescent cells holds great promise for cancer treatment-related and senescence-associated bone disorders.

## Graphical abstract

**Figure:**
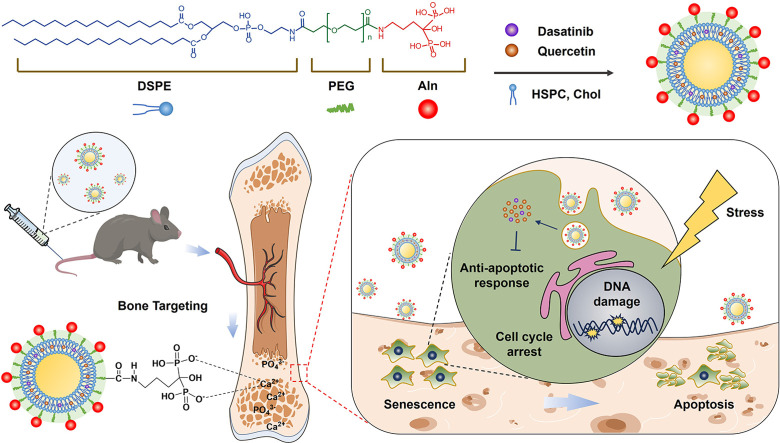

## 1 Introduction

Osteoporosis is a prevalent metabolic bone disorder affecting the entire skeletal system, characterized by decreased bone density, weakened bone integrity, and impaired bone microarchitecture [1,2]. Osteoporosis increases the risk of fractures, reduces quality of life, and leads to disability and mortality [2,3]. Various etiologies result in osteoporosis, and postmenopausal and age-related bone loss are the most common clinical subtypes [4,5]. Other risk factors for osteoporosis, such as long-term use of glucocorticoids, radiotherapy and chemotherapy, are defined as secondary osteoporosis [6,7]. Various treatments have been explored for different etiologies of osteoporosis, including denosumab, bisphosphonates, calcitonin, and selective estrogen receptor modulators. Nonetheless, these therapeutic options demonstrate limited efficacy or necessitate prolonged administration accompanied by adverse effects. It is worth noting that individuals diagnosed with both cancer and osteoporosis experienced early onset of bone metastasis compared to those without osteoporosis [8,9]. This underscores the critical need for safe and effective therapeutic strategies tailored for osteoporosis patients, particularly those concurrently managing other conditions such as cancer.

Cellular senescence is considered one of the key contributors to bone loss [10] and has been proposed as a promising target for osteoporosis due to its pivotal role during bone remodeling [[11], [12], [13], [14]]. For example, younger bone marrow mesenchymal stem cells (BMMSCs) exhibited a balanced osteogenic-lipogenic capacity [15]. In contrast, senescent BMMSCs showed a reduced osteogenic and enhanced lipogenic capacity, which led to final bone loss with senescence [[16], [17], [18]]. This imbalance in bone remodeling ultimately contributes to the development of osteoporosis. Moreover, the accumulation of senescent cells in the bone tissues increases senescence-associated secretory phenotype (SASP) secretion. The SASP can induce a pro-inflammatory microenvironment that promotes the survival and differentiation of osteoclast progenitor cells [11,12]. Thus, bone loss after cancer treatments is possibly due to chemotherapy or radiotherapy-induced BMMSC senescence which disrupts the balance of normal bone metabolism [19,20].

Previous studies have demonstrated that either eliminating senescent cells or inhibiting the secretion of pro-inflammatory factors can prevent cellular senescence-related bone loss [[21], [22], [23], [24], [25]]. Senolytic agents, a combination of dasatinib (D) and quercetin (Q), can selectively eliminate senescent cells that cause tissue damage, and clinical trials have substantiated their efficacy in this regard [[26], [27], [28]]. Notably, when administered at optimal concentrations and ratios, D and Q can effectively induce apoptosis in senescent cells while exerting minimal impact on normal cells [29]. Additionally, these senolytic drugs have the potential to reduce the load of senescent cells and enhance the osteogenic ability of senescence BMMSCs [30], while also reducing the generation of osteoclasts, thereby inhibiting bone resorption and promoting bone formation or maintenance [31,32]. As a result of this mixed anti-resorptive/anabolic effect, D and Q-based senolytics therapy may confer more enduring benefits on bone health than conventional anti-resorptive therapies [12,33].

However, due to the poor water solubility of D and Q, their bioavailability after oral administration is limited, which in turn affects drug penetration efficiency into target tissues [34,35]. Furthermore, the lack of specificity for particular cell populations or tissues may lead to systemic side effects when the local therapeutic dose is achieved [[35], [36], [37]]. These issues impair the potential therapeutic efficacy and may lead to side effects. Additionally, attaining the accurate concentrations of both drugs at the same sites also met difficulties. Hence, bone-targeted delivery of senolytics could achieve the targeted release of senolytic drugs at the same time with a precise ratio of drugs. The bone targeting could be achieved by alendronate owing to its high affinity for bone minerals [[38], [39], [40]]. This strategy can effectively improve the bioavailability of senolytics drugs, which can be targeted and synergistically administered to reduce the enrichment of senescent cells locally in the bone tissue, thereby reducing bone loss and slowing down the process of osteoporosis.

## 2 Materials and methods

### 2.1 Materials

1,2-distearoyl-sn‑glycero-3-phosphoethanolamine-N-[hydroxysuccinimidyl-(polyethylene glycol)−2000] (DSPE-PEG-NHS, Aladdin, China), Hydrogenated soya phosphatidylcholines (HSPC, Nippon Fine Chemical, Japan), Cholesterol (Chol, J&K, China), dasatinib (D, Macklin, China), quercetin (Q, Macklin, China), Alendronate (Aln, Heowns, China).

### 2.2 Experimental mouse models

Mice (C57BL/6, male, 8-week-old) were purchased from Vital River Laboratory Animal Technology Co. Ltd. (Beijing, China). To induce osteoporosis, two distinct models were established: chemotherapy-induced and radiotherapy-induced. The chemotherapy-induced model was established as previously reported, in which mice were administered doxorubicin (Dox) at 5 mg/kg by intraperitoneal injection [10]. This treatment was administered once a week for a continuous period of 4 weeks to mimic the effects of chemotherapeutic agents on bone health. At the same time, mice in the vehicle group were given an equivalent volume of PBS. In the radiotherapy-induced model, the mice were anesthetized and positioned under an irradiation (IR) apparatus, with the lower limbs exposed to a radiation dose of 15 Gy. At the same time, the rest of the body was shielded with a plate to confine the radiation effect of lead.

### 2.3 Synthesis and characterization of DSPE-PEG-Aln

DSPE-PEG-Aln was synthesized by conjugated alendronate to DSPE-PEG-NHS via a conjugating reaction between amine and NHS ester [41]. Briefly, DSPE-PEG-NHS (34.0 mg, 0.01 mol) and alendronate (19.5 mg, 0.06 mol) were added into PBS at pH 8 (10 mL), stirring at room temperature for 24 h The obtained solution was collected in a dialysis bag (1000 Da MWCO) and dialysis for 72 h. The final product was obtained by lyophilization and characterized by nuclear magnetic resonance (NMR) spectroscopy.

### 2.4 Preparation and characterization of Aln-Lipo-DQ

The preparation of Lipo-DQ and Aln-Lipo-DQ is conducted by the thin-film hydration method. Firstly, Aln-Lipo-DQ was prepared as follows: D: Q: HSPC: DSPE-PEG-Aln: DSPE-PEG_2000_: Chol: = 0.008: 1.2: 30: 4: 2: 1 (w: w) were dissolved in chloroform-methanol (4:1) mixture. Then, the organic solvent was rotationally evaporated under vacuum conditions. After a yellow film appeared at the bottom of the flask it was hydrated with PBS (pH 7.4) and sonicated for 10 min to obtain crude liposomes. The crude liposomes were repeatedly extruded with 400 nm and 200 nm polycarbonate membranes successively and dialyzed in PBS overnight to remove the unencapsulated free *D* + *Q*. Lipo-DQ was prepared by replacing all DSPE-PEG-Aln with DSPE-PEG_2000_. The particle size and zeta potential of the liposomes were characterized by dynamic light scattering, and their morphology was observed using transmission electron microscopy (TEM). The liposome solutions were examined by measuring their particle size and PDI at 0, 1, 2, 6, 24, and 48 h at room temperature for their stability. For the serum stability assessment of the liposomes, they were first mixed with 10% fetal bovine serum and then incubated at 37 °C. DLS measurements were taken at various time points over a 24 h period. Cyanine 5.5 (Cy5.5) (1% of total lipids, w: w) was mixed with the liposome constituents for fluorescent labeling.

### 2.5 Hydroxyapatite (HAp) binding assay

To assess the *in vitro* bone-binding capacity of Aln-Lipo, various concentrations of Aln-Lipo and Lipo (0.1, 0.5, 1.0 mg/mL) loaded with Cy5.5 were incubated with HAp at room temperature for 4 h After incubation, the samples were centrifuged, and the supernatant was separated from the HAp precipitate. Fluorescence imaging and spectroscopy were then used to measure the supernatant containing Aln-Lipo-Cy5.5 and Lipo-Cy5.5-treated HAp.

### 2.6 *In vivo* bone-targeting and tissue distribution

Mice were intravenously administered with 200 µL of Lipo-Cy5.5 and Aln-Lipo-Cy5.5 liposome solutions (5 mg/mL). After 24 h, organs, including the heart, liver, spleen, lungs, and kidneys, were collected. The distribution intensity of the drugs in each tissue was evaluated using an in *vivo* imaging system (IVIS). For bone tissue, the fluorescence intensity of the femur and tibia was detected at 1 and 7 days post-injection.

### 2.7 Primary BMMSCs cell culture

Primary BMMSCs are isolated from mice and cultured as previously described. Mice’s tibia and femur were dislocated, and the muscle and connective tissue on them were removed under aseptic conditions. Then, the bone slices, with the bone marrow matrix removed, were divided into smaller pieces and incubated with type II collagenase at 37 °C for 2 h. The bone slices and cells were collected by centrifuge, then seeded in the α-MEM complete medium supplemented with 10% fetal bovine serum. These cultures were maintained at 37 °C and 5% CO_2_ in a humidified incubator and replenished medium every three days. Cells were cultured in the medium containing 100 nM dexamethasone after one week. BMMSCs were passaged after growth to 80%, and subsequent experiments were performed in passages 2–3.

### 2.8 Establishment of senescent BMMSC models

Normal BMMSCs from 8-week-old C57BL/6 mice were seeded in multi-well plates or culture dishes at an appropriate density. For Dox-induced senescence, the cells were exposed to 0.2 µM Dox for 24 h after adhesion. After an additional 3 days of cultivation, the cells were prepared for *in vitro* experiments. After adhesion, the cells were subjected to a 10 Gy irradiation dose for radiotherapy-induced senescence. The culture medium was refreshed on the second day, and the cells were then incubated for another 5 days before use.

### 2.9 Drug administration

Osteoporotic mice were randomly divided into four groups for detailed deployments of animal experiments. (1) naïve group: the untreated mice were *iv.* injected with 200 µL PBS; (2) Dox + PBS or IR + PBS group: The model group was administered intravenously 200 µL of PBS; (3) Dox + DQ or IR + DQ group: The conventional administration group was treated with 5 mg/kg D and 50 mg/kg Q orally to osteoporotic mice; (4) Dox + Lipo-DQ or IR + Lipo-DQ group: 200 µL of nanoliposome suspension (Lipo-DQ, containing 5 mg/kg *D + Q*) was administered intravenously to osteoporotic mice as the control group for bone-targeted liposome treatment; (5) Dox + Aln-Lipo-DQ or IR + Aln-Lipo-DQ group: 200 µL nanoliposome suspension (Aln-Lipo-DQ, containing 5 mg/kg *D + Q*) was administered intravenously to osteoporotic mice as a bone-targeting *D* + *Q* in the treatment group.

### 2.10 Micro-computed tomography (µCT) analysis

First, the femurs were placed vertically in 12-mm scanning tubes. The samples were then scanned for distal femoral epiphyses using micro-computed tomography (µCT) (Bruker, Skyscan 1275). The scanning parameters were set to 50 kV and 200 µA, with an image resolution of 6 µm per pixel. After scanning, we chose the target region, which was defined as a 200-slice thickness starting from 150 slices below the growth plate, to assess bone mass. The data were further processed using CTAn software to analyze parameters such as trabecular bone volume per tissue volume (BV/TV; %), trabecular number (Tb.N; 1/mm), trabecular thickness (Tb.Th; mm) and trabecular separation (Tb.Sp; mm). Additionally, 3D image reconstruction was further performed using CTvox software.

### 2.11 Histomorphometric analyses

Briefly, the femoral tissues underwent dehydration and decalcification processes before being embedded in paraffin for further analysis. TRAP activity staining was used to evaluate the osteoclasts *in vivo*. The immunofluorescence staining procedure was conducted as follows: Initially, the bone sections were first dewaxed in xylene and then rehydrated in distilled water. The sections were incubated in a 1% solution of Triton X-100 to enhance permeability, followed by antigen retrieval with an antigen retrieval solution and blocking with a 3% BSA solution (Servicebio, China). Primary antibodies of Ocn (1:400), p16 (1:400), and p21 (1:400, Abcam, USA) were incubated overnight at 4 °C. On the following day, a secondary antibody (Cy3-labeled goat anti-rabbit, 1:400, Servicebio, China) was applied for 60 min at room temperature, away from light. DAPI (Beyotime, China) was used to stain cell nuclei. Fluorescence images were captured with a digital slice scanner (3DHISTECH, Hungary). The protein expression levels of Ocn, p16, and p21 were quantified through the enumeration of red-fluorescent pixels, indicative of positive cellular regions, utilizing the ImageJ software.

### 2.12 Statistical analyses

All results are expressed as mean ± SD. Statistical analyses were conducted using GraphPad Prism 8 (GraphPad software). Statistical significance was assessed using Student's *t*-test for comparisons between two groups and ANOVA for comparisons among multiple groups. A significance level of *P < 0.05 was considered significant, **P < 0.01, ***P < 0.001, and ****P < 0.0001 indicated progressively higher levels of statistical significance.

## 3 Results

### 3.1 Fabrication and characterization of Aln-Lipo-DQ

**Fig. 1:**
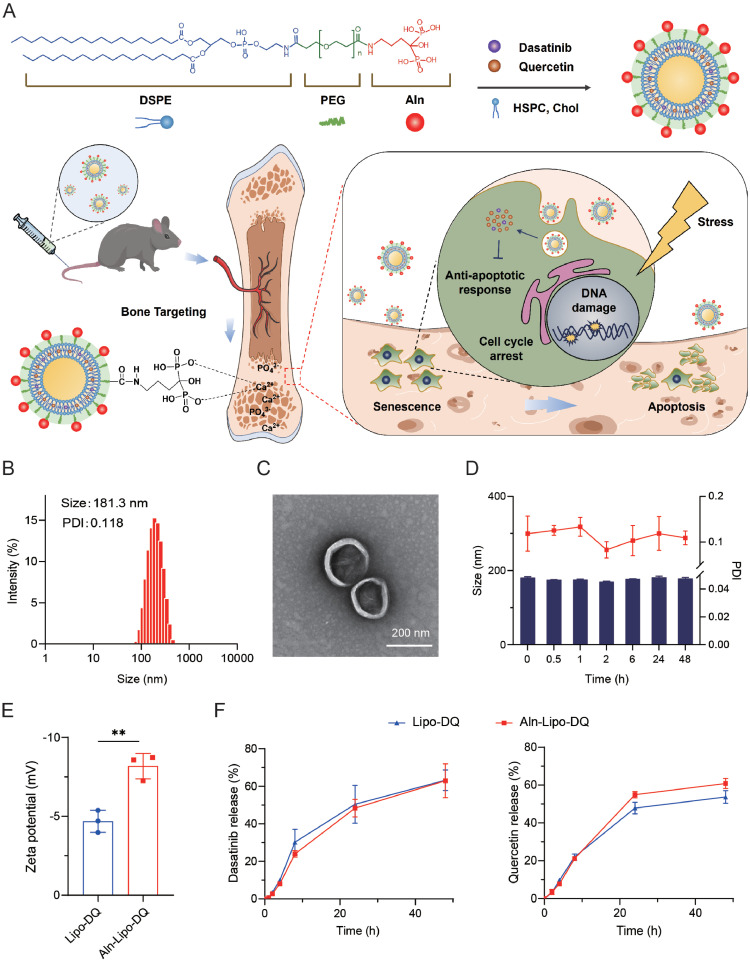
**Fabrication and characterization of Aln-Lipo-DQ.** (A) Schematic depiction of liposomes modified with alendronate encapsulates senolytic drugs for the targeted delivery to osseous tissue to eliminate senescent cells. (B) The size and distribution of Aln-Lipo-DQ were analyzed by DLS. (C) TEM visualizes the structure of Aln-Lipo-DQ. (D) Stability of Aln-Lipo-DQ at room temperature for 48 h (E) The zeta potential of Aln-Lipo-DQ and Lipo-DQ. (F) *In vitro* dasatinib and quercetin release in PBS (pH 7.4) containing 1% Tween 80 at 37 °C within 48 h. **P < 0.01.

Alendronate (Aln) has two phosphate groups that can form chelation complexes with Ca^2+^. Thus, Aln has a high binding affinity to hydroxyapatite (HAp), the bone matrix's main inorganic constituent [38,41]. For targeted clearance of senescent cells in bone tissue, anti-senescent drugs D and Q are encapsulated by Aln-modified liposomes (Fig. 1A). DSPE-PEG-NHS (DPN) was used to conjugate Aln, with the NHS group facilitating bond formation with Aln's amine group (Fig. S1A). The DSPE-PEG-Aln (DPA) was characterized by NMR hydrogen spectroscopy. The nuclear magnetic signal peaks for -NCH_2_- and -CH_2_CH_2_C- from alendronate at 1.91 ppm and 2.95 ppm, respectively, were observed in the spectrum of DPA. Additionally, the signal peaks of DPN at 2.85 ppm (corresponding to -COCH_2_CH_2_CO-) disappeared in the spectrum of DPA. The signal peaks associated with the -NCH_2_- group in the Aln moiety and the -CH_3_ group in the DSPE moiety within the synthetic product were analyzed further. These results demonstrated the successful synthesis of DPA, with a yield of 84.26% (Fig. S1B).

The bone-targeting liposomes loaded with D and Q (Aln-Lipo-DQ) were then prepared using DPA, whereas control liposomes (Lipo-DQ) lacked this lipid. The liposome’s particle size and zeta potential were determined via dynamic light scattering (DLS). The measured particle size of Aln-Lipo-DQ was 181.3 nm, exhibiting a favorable polydispersity index (PDI) (Fig. 1B). Transmission electron microscopy (TEM) images corroborated the spherical morphology of the liposomes, with a particle size of approximately 200 nm (Fig. 1C). Similarly, the measured particle size of Lipo-DQ was 183.7 nm and exhibited a spherical structure (Fig. S2). DLS further tested the stability of the Aln-Lipo-DQ both at room temperature and in serum. As shown in Fig. 1D, there were no significant particle size or PDI alterations over time after storage at room temperature. Furthermore, when incubated in 10% fetal bovine serum, DLS measurements indicated that Aln-Lipo-DQ maintained stable particle size and PDI 24 h (Fig. S3). Meanwhile, due to the incorporation of phosphate groups from alendronate, the zeta potential of the liposome was observed to turn more electronegative (Fig. 1E). We also evaluated the drug loading capacity and drug release of Aln-Lipo-DQ *in vitro*. The samples were lyophilized and measured by HPLC, and the encapsulation rate of D and Q in Aln-Lipo-DQ was obtained as 94.04% and 94.53%, respectively. Subsequently, the *in vitro* drug release from Aln-Lipo-DQ was investigated under sink conditions using dialysis. The results showed that D and Q in liposomes were slowly released up to 60% within 48 h (Fig. 1F). The involvement of alendronate does not affect the drug release of liposomes, and the encapsulation of liposomes can achieve sustained drug release.

### 3.2 Aln-Lipo exhibiting superior binding affinity to HAp *in vitro*

**Fig. 2:**
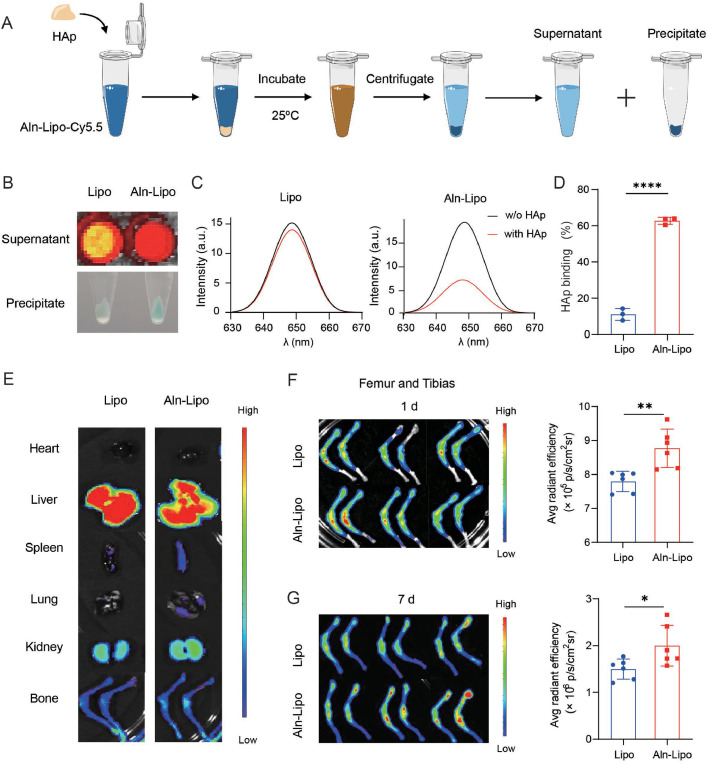
**Aln-Lipo exhibited bone targeting capacity both *in vitro* and *in vivo.*** (A) Schematic illustration of the binding capacity of Aln-Lipo to HAp *in vitro*. (B) The NIR images and photographs of supernatant and HAp precipitation, respectively. (C) The fluorescence spectrum of supernatant of HAp after treatment with Aln-Lipo-Cy5.5 and Lipo-Cy5.5, respectively. (D) HAp binding affinity of Aln-Lipo-Cy5.5 and Lipo (*n* = 3). (E) The *ex vivo* images of excised major organs at 24 h after injection of Aln-Lipo-Cy5.5 or Lipo-Cy5.5. (F, G) The *ex vivo* images of femur and tibias at 1 d or 7 d injection of Aln-Lipo-Cy5.5 or Lipo-Cy5.5 and semi-quantitative NIR analysis by IVIS (*n* = 6). *P < 0.05, **P < 0.01 and ****P < 0.0001.

Aln-Lipo was labeled with Cy5.5 to verify the bone targeting capacity *in vitro*. Firstly, both Aln-Lipo-Cy5.5 and Lipo-Cy5.5 were incubated with HAp at room temperature at varying concentrations according to previous report with slight change [39] (Fig. 2A). The results showed that the fluorescence signal in the supernatant for each concentration of Aln-Lipo was significantly lower than that of Lipo (Figs. 2B and S4), suggesting that Aln-Lipo binds more effectively to HAp. Given that Cy5.5 appears blue in natural light when incorporated into liposomes, the binding of Aln-modified liposomes to HAp caused the HAp crystals to turn blue. As shown in the figure, with the increase in liposome concentration, the HAp precipitate of Aln-Lipo in the centrifuge tube deepened blue, whereas the HAp precipitate for Lipo remained white (Figs. 2B and S4). The binding affinity of liposomes to HAp was indirectly measured by assessing the fluorescence intensity of the supernatant using a fluorescence spectrophotometer. The result showed that the HAp binding rate of Lipo was 11.09%, while Aln-Lipo exhibited a significantly higher binding rate of 62.69% (Fig. 2C, D). From this, it can be seen that Aln-Lipo has potential bone-targeting ability.

### 3.3 *In vivo* biodistribution of Aln-Lipo

The *in vivo* bone-targeting capability of the Aln-Lipo was further examined by assessing its biodistribution following systemic infusion. Mice were intravenously injected with Lipo-Cy5.5 and Aln-Lipo-Cy5.5 liposome solutions, and the major organs (heart, liver, spleen, lung, kidney, bone) were collected 24 h post-injection. Drug distribution within these tissues was analyzed using an IVIS. The results revealed that both liposome formulations accumulated predominantly in vascular-rich organs (liver, spleen, and kidneys) (Fig. 2E). Notably, imaging of the femur and tibia at 1 and 7 days post-injection showed significantly higher fluorescence intensity in bone tissue for the Aln-Lipo group than the Lipo group. These findings suggest that Aln-modified liposomes serve as an effective drug delivery system for the targeted delivery of *D* + *Q* to bone tissue (Fig. 2F, G).

### 3.4 Liposomal dasatinib and quercetin synergistically eliminate senescent BMMSCs

Initially, primary culture BMMSCs were isolated from mice through bone chip digestion and further purified. Surface markers on the purified cultured cells were analyzed by flow cytometry to confirm the successful isolation of BMMSCs. The results showed that the cells with high expression of Sca-1, CD44, and CD29, with negligible expression of CD34 and CD45 (Fig. S5), demonstrated successful BMMSCs isolation [42].

**Fig. 3:**
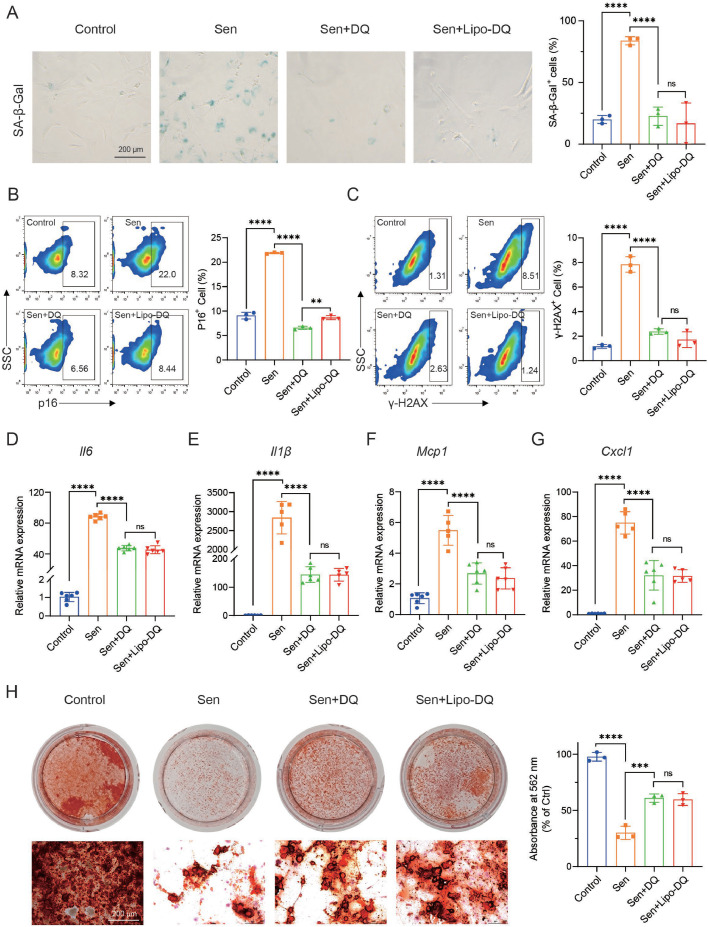
**Liposomal dasatinib and quercetin synergistically eliminate senescent BMMSCs.** (A) The representative images and percentage of SA-β-Gal^+^ cells were used to evaluate cellular senescence (*n* = 3). (B, C) The proportion of p16^+^ and γ-H2AX^+^ cells after receiving *D* + *Q* or Lipo-DQ treatment was analyzed by flow cytometry, respectively (*n* = 3). (D-G) Relative mRNA expression levels of *Il6, Il1β, Cxcl1, and Mcp1* in senescent BMMSCs after *D* + *Q* or Lipo-DQ treatment (*n* = 5–6). (H) Representative images of alizarin red staining and semi-quantitative analysis of senescent BMMSCs treated by *D* + *Q*, Lipo-DQ (*n* = 3). ***P < 0.001 and ****P < 0.0001.

Cellular senescence of BMMSCs was induced by doxorubicin (Dox) and characterized by assessing SA-β-Gal staining and the expression of both p16^Ink4a^ (p16) and γ-H2AX. The results indicated that 0.2 µM Dox could effectively induce the senescence of BMMSCs (Fig. 3A, B). The viability of normal BMMSCs after being treated with dasatinib, quercetin, and a combination of both at varying concentrations was evaluated. Dasatinib at 0.1 µM and quercetin at 20 µM were selected for subsequent experiments to balance the efficacy and toxicity (Fig. S6). To determine whether the senolytics combination (*D* + *Q*) could selectively eliminate senescent BMMSCs with negligible effects on normal BMMSCs, they were separately treated on normal and senescent BMMSCs. The crystal violet staining shows that senolytics (*D* + *Q*) were significantly more toxic to senescent BMMSCs than to normal counterparts. Notably, the liposomal senolytics showed even more toxicity on senescent BMMSCs (Fig. S7). Additionally, Aln-Lipo alone was then applied to normal BMMSCs to assess its potential cytotoxicity. The results showed that no cytotoxic effect was observed at concentrations ranging from 0.03125 to 1.000 mg/mL (Fig. S8).

The targeted elimination capacity of both *D* + *Q* and Lipo-DQ on senescent BMMSCs was evaluated. The SA-β-Gal staining showed that senescent BMMSCs exhibited fewer SA-β-Gal positive cells after both *D* + *Q* and Lipo-DQ treatment (Fig. 3A). Meanwhile, the expression of p16 and the DNA damage marker γ-H2AX in senescent BMMSCs were also significantly decreased (Fig. 3B, C). SASPs were considered a hallmark of cellular senescence. Then, we evaluated the mRNA expression of several typical SASPs, which are secreted by senescent BMMSCs and could negatively affect normal BMMSCs function through paracrine signaling. The analysis revealed that treatment with *D* + *Q* and Lipo-DQ could significantly reduce the expression of various inflammatory mediators associated with senescent BMMSCs, including *Il6, Il1β, Cxcl1, and Mcp1* (Fig. 3D-G). In addition to cellular senescence, the treatment also improved the osteogenic induction of senescent BMMSCs. The alizarin red staining was performed at the end of 21 days of osteogenic induction. A remarkedly increase in calcium nodule production after *D* + *Q* and Lipo-DQ treatment was observed in contrast to the untreated group (Fig. 3H). All of these results demonstrate that senolytics (*D* + *Q*) and Lipo-DQ can reduce the senescence burden and restore the proliferative capacity and osteogenic differentiation potential of senescent BMMSCs by targeted elimination of senescent BMMSCs. In addition, it was shown that similar trends were also present in radiotherapy-induced senescent BMMSCs, mirroring the results seen with Dox-induced senescence (Fig. S9, 10).

### 3.5 Targeted clearance of senescent cells in bone tissue alleviates chemotherapy-induced osteoporosis

**Fig. 4:**
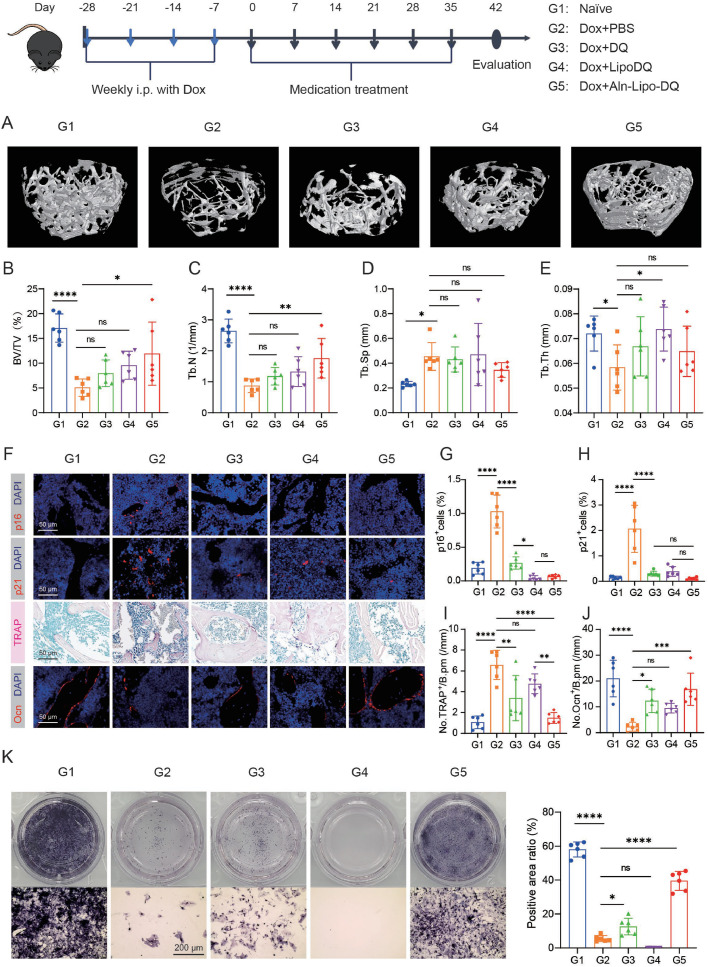
**Targeted clearance of senescent cells in bone tissue alleviates chemotherapy-induced osteoporosis.** (A) Micro-CT (µCT) 3D images of the femur in each treatment group. Quantification of µCT-derived (B) bone volume fraction (BV/TV; %), (C) trabecular number (Tb.N; 1/mm), (D) trabecular separation (Tb.Sp; mm), (E) trabecular thickness (Tb.Th; mm). Immunofluorescent images of p16 (F, G) and p21 (F, H) expression in the femur with quantification (*n* = 6, scale bar is 50 µm). (F, I) Representative images of TRAP (osteoclast marker) staining and quantification analysis in femurs (*n* = 6, scale bar is 50 µm). (F, J) Immunofluorescent images of osteocalcin (Ocn, mature osteoblast marker) expression in the femur with quantification (*n* = 6, scale bar is 50 µm). Cells stained positively for markers are quantified as numbers per bone perimeter (B.pm). (K) ALP staining of BMMSCs from each treatment group after 14 days of osteogenic induction (*n* = 6, scale bar is 200 µm). *P < 0.05, **P < 0.01, ***P < 0.001 and ****P < 0.0001.

We explore the efficacy of Aln-Lipo-DQ in the treatment of chemotherapy-induced bone loss in mice. First, 8-week-old healthy male mice (C57BL/6) were injected with Dox for 4 weeks to induce bone senescence, followed by treatments with PBS, *D* + *Q*, Lipo-DQ, and Aln-Lipo-DQ, respectively. After treatment, micro-CT scans were performed on the femurs of each group. 3D modeling imaging showed that Dox-treated mice developed severe bone loss, which was improved after drug treatment. Compared with the *D* + *Q* and Lipo-DQ groups, the Aln-Lipo-DQ treatment had better trabecular bone microstructure (Fig. 4A). Further quantitative analysis of bone-related indices such as trabecular bone volume fraction (BV/TV), number (Tb.N), segregation (Tb.Sp), and thickness (Tb.Th) showed that drug-treated mice had better bone-related indices compared to the model group (Fig. 4B-E). Although no statistically significant differences were observed between the drug-treated groups, the Aln-Lipo-DQ treatment group demonstrated a notable improvement in Dox-induced bone loss (Fig. 4B-E).

To further investigate the regulation of Aln-Lipo-DQ on the bone microenvironment, we performed immunofluorescence staining for p16 and p21 on senescent cells within the bone tissue. Our findings revealed a significant increase in the number of p16^+^ and p21^+^ cells in the bone tissue of Dox-treated mice than untreated mice (Fig. 4F-H). Treatment with the drug significantly reduced the number of Dox-induced senescent cell accumulation (Fig. 4F-H). We then performed a quantitative analysis of osteoblasts and osteoclasts in the trabecular bone. The TRAP staining showed that both *D* + *Q* and Aln-Lipo-DQ significantly reduced the Dox-induced osteoclast increase (Fig. 4F, I). Immunofluorescence results of the osteoblast marker Ocn demonstrated an increase in Ocn expression in drug-treated mice compared to the PBS treatment group, with the Aln-Lipo-DQ group showing a significant increase in the number of osteoblasts (Fig. 4F, J).

We also isolated BMMSCs from each experimental group to assess their osteogenic potential. After culturing in an osteogenic medium for 14 days, we performed ALP staining, which is an early biomarker of osteoblast differentiation. The results showed that the Aln-Lipo-DQ group’s BMMSCs had a better potential to differentiate into osteoblasts than other treatment groups (Fig. 4K). Additionally, colony formation assays demonstrated that *D* + *Q* and Aln-Lipo-DQ group’s BMMSCs had a significantly higher number of colonies than the model group’s (Fig. S11A). These results indicated that Aln-Lipo-DQ effectively removed the senescent cells in bone tissue, leading to a notable increase in osteoblasts involved in bone formation. At the same time, this treatment significantly reduces the number of osteoclasts, which are critical for bone resorption, thereby greatly improving the bone microenvironment necessary for maintaining bone homeostasis. Furthermore, Aln-Lipo-DQ enhances the functionality and metabolic activity of BMMSCs in Dox-treated mice, potentially increasing the regenerative capacity of the skeletal system.

### 3.6 Targeted clearance of senescent cells in bone tissue alleviates radiotherapy-induced osteoporosis

**Fig. 5:**
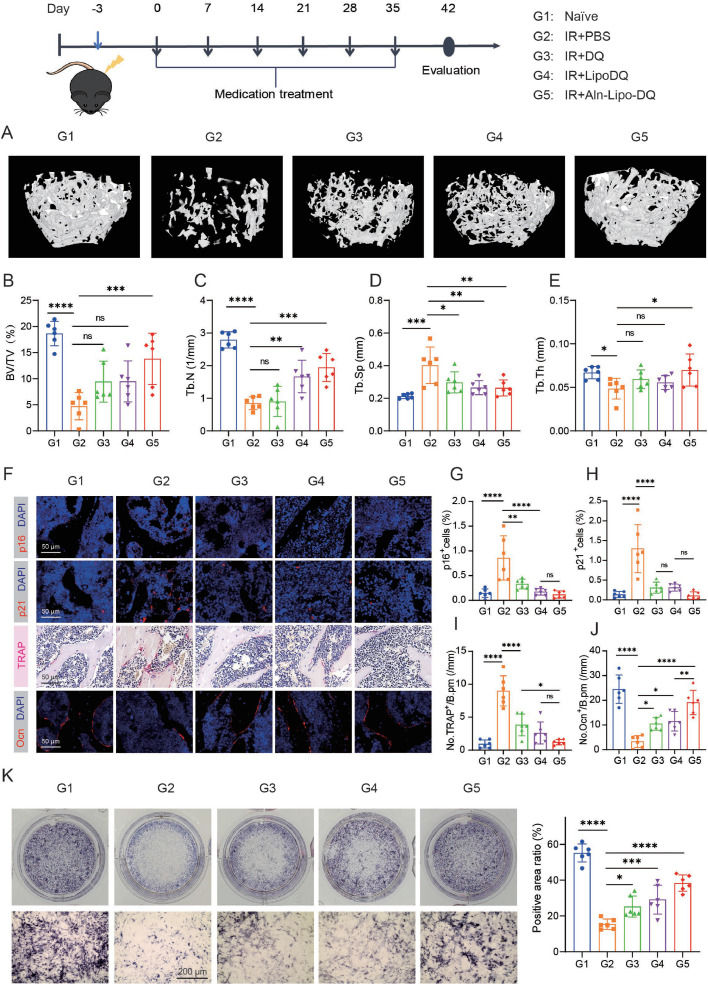
**Targeted clearance of senescent cells in bone tissue alleviates radiotherapy-induced osteoporosis.** (A) Micro-CT (µCT) 3D images of the femur in each treatment group. Quantification of µCT-derived (B) bone volume fraction (BV/TV; %), (C) trabecular number (Tb.N; 1/mm), (D) trabecular separation (Tb.Sp; mm), (E) trabecular thickness (Tb.Th; mm). Immunofluorescent images of p16 (F, G) and p21 (F, H) expression in the femur with quantification (*n* = 6, scale bar is 50 µm). (F, I) Representative images of TRAP staining and quantification analysis in femurs (*n* = 6, scale bar is 50 µm). (F, J) Immunofluorescent images of Ocn expression in the femur with quantification (*n* = 6, scale bar is 50 µm). Cells stained positively for markers are quantified as numbers per bone perimeter (B.pm). (K) ALP staining of BMMSCs from each treatment group after 14 days of osteogenic induction (*n* = 6. scale bar is 200 µm). *P < 0.05, **P < 0.01, ***P < 0.001 and ****P < 0.0001.

We also examined Aln-Lipo-DQ for the treatment of radiation-induced bone loss in mice. 8-week-old healthy male mice (C57BL/6) were subjected to a 15 Gy irradiation, which caused a marked increase in senescent cells expressing p16 and p21 within the bone, along with a marked reduction in the osteogenic capacity of BMMSCs, ultimately leading to severe bone loss. Drug treatment was initiated immediately after radiation, and femoral parameters were assessed at the end of the treatment period. The results showed that the Aln-Lipo-DQ group exhibited significantly improved bone parameters in the radiotherapy models, including a 2.91-fold increase in bone volume fraction compared to the control (Fig. 5A, B), as well as significantly higher trabecular number (Fig. 5C) and thickness (Fig. 5E), along with a notable improvement in trabecular separation (Fig. 5D). Similar to its effects on Dox-induced bone loss, Aln-Lipo-DQ effectively cleared senescent cells from bone tissue (Fig. 5F-H), increased the number of osteoblasts (Fig. 5F, I), declined the number of osteoclasts (Fig. 5F, J), and also ameliorated the radiation-induced decrease in cell activity and osteogenic differentiation capacity of BMMSCs (Figs. 5K and S11B). Furthermore, we assessed the *in vivo* biosafety of Aln-Lipo-DQ. Hematoxylin and eosin (H&E) staining of major organ tissues from the *D* + *Q*, Lipo-DQ, and Aln-Lipo-DQ treatment groups showed no histopathological abnormalities or lesions compared to the control group (Fig. S12), indicating favorable biocompatibility and safety for Aln-Lipo-DQ.

## 4 Discussion and conclusion

In this study, we have demonstrated that senolytics combination (*D* + *Q*) could be efficiently encapsulated into Aln-Lipo, and alendronate modification could significantly improve the bone affinity of liposomes both *in vitro* and *in vivo*. Additionally, we confirmed that senolytics (*D* + *Q*) could specifically target and eliminate senescent BMMSCs, thereby enhancing the activity and osteogenic potential of senescent BMMSCs *in vitro*. Considering the clinical efficacy of dasatinib and quercetin is often constrained by poor bioavailability and off-target effects in non-osseous tissues, our alendronate-functionalized liposomes (Aln-Lipo-DQ), capitalizing on alendronate’s high affinity for bone minerals, could achieve precise delivery of senolytic directly to bone tissue. This dual-targeting approach not only improves the bioavailability of senolytics at the intended site but also minimizes off-target effects, thereby enhancing the overall safety and efficacy of the treatment. Notably, consistent findings *in vivo* also confirm that Aln-Lipo-DQ is more effective in clearing senescent cells from bone tissue compared to oral *D* + *Q* and Lipo-DQ, leading to improved BMMSC activity, reduced bone resorption, and increased bone formation. In contrast to traditional anti-resorbing therapy, the senolytic approach could overcome the coupling between osteoblasts and osteoclasts, which often reduces bone formation while inhibiting resorption and can compromise therapeutic efficacy [43]. Besides, applying targeted delivery carriers could further improve the efficacy of senolytics by overcoming the low perfusion and the lack of capacity to cross the endothelial cells into the bone microenvironment. Therefore, this dual-targeting strategy aimed at senescence presents a promising method for the clinical management of bone loss and offers an effective therapeutic option for patients with therapy-induced osteoporosis.

Tumor patients normally undergo multiple therapies, like chemotherapy or radiotherapy, which cause the accumulation of senescent cells. Recent studies suggest a potential link between cellular senescence and the development of bone metastasis in cancer patients. The accumulation of senescent cells, along with the resulting bone resorption, releases a variety of inflammatory factors that stimulate tumor cell growth and metastasis [44,45]. Intervening in the p38MAPK/MK2 pathway, which mediates the secretion of SASP, has been shown to limit chemotherapy-induced bone loss and breast cancer metastasis [46]. Therefore, targeting the elimination of senescent cells not only improves osteoporosis in patients but also reduces the potential for bone metastasis. Moreover, the inflammatory microenvironment induced by cancer therapies can further impact drug absorption [[47], [48], [49]]. As such, bone-targeted drug delivery systems play a crucial role in this complex therapeutic environment [50,51]. The incorporation of nanotechnology enhances drug stability, bioavailability, and targeting precision by encapsulating drugs in nanocarriers, facilitating more targeted and efficient delivery [[52], [53], [54], [55]]. Additionally, by minimizing systemic drug exposure, these systems reduce the risk of adverse effects on other organs and tissues, as well as decrease the potential for drug interactions, thereby improving both treatment safety and efficacy [56,57]. Thus, targeted delivery senolytics to modulate the bone microenvironment may provide a promising strategy for cancer patients.

## Ethics approval statement

All animal experiments were performed in accordance with animal use protocols approved by the Committee for the Ethics of Animal Experiments, the Institutional Animal Care and Use Committee of the National Center for Nanoscience and Technology (approval number: NCNST-LX-2203-46).

## Data and materials availability

All data related to this study are presented in the paper or the supplementary materials.

## Declaration of competing interest

The authors declare that they have no conflicts of interest in this work.

## Appendix Supplementary materials

Image, application 1
