# Supplementary material for: Targeted delivery of liposomal senolytics to alleviate cellular senescence-induced bone loss
Source: Fundam Res. 2024 Dec 27;5(4):1429–39. doi: 10.1016/j.fmre.2024.12.010 (PMC12327876; doi:10.1016/j.fmre.2024.12.010)
Supplement: Supplementary file 1 [file mmc1.docx]

Supporting Information for

**Targeted Delivery of Liposomal Senolytics to Alleviate Cellular Senescence-Induced Bone Loss**

Rong Li^a,b,1^ , Yaohua Wei^b,1^, Changhao Xiong^b^, Jingwei Wang^b,d^, Yixuan Lin^b^, Ronghui Deng^b^, Hao Qin^b^, Yang Chen^b^, Nan Li^b^, Guyu Zheng^b^, Yuanyuan Lv^b^, Jian Shi^b^, Tingting Yu^d^, Yiye Li^b,c^, Jing Wang^e^, Ruifang Zhao^b,^^c,^*, Changsheng Liu^e,^*, Guangjun Nie^b,c,^*

^a^ College of Chemistry, Zhengzhou University, Zhengzhou, 450001, P. R. China

^b^ CAS Key Laboratory for Biomedical Effects of Nanomaterials and Nanosafety, CAS Center for Excellence in Nanoscience, National Center for Nanoscience and Technology, Beijing, 100190, P. R. China

^c^ Center of Materials Science and Optoelectronics Engineering, University of Chinese Academy of Sciences, Beijing, China

^d^ Department of Orthodontics, Peking University School and Hospital of Stomatology, Beijing, China

^e^ Key Laboratory for Ultrafine Materials of Ministry of Education, East China University of Science and Technology, Shanghai, 200237, China

^1^These authors contributed equally to this work.

*Corresponding authors.

E-mail addresses: [zhaorf@nanoctr.cn](mailto:zhaorf@nanoctr.cn) (Z. Zhao); [liucs@ecust.edu.cn](mailto:liucs@ecust.edu.cn) (L. Liu); [niegj@nanoctr.cn](mailto:niegj@nanoctr.cn) (G. Nie)

**Methods**

**Drug-loading efficiency and drug release**

Lipo-DQ and Aln-Lipo-DQ samples were freeze-dried to obtain yellow powder. A certain amount was dissolved in methanol solution, thoroughly dissolved, and then filtered through a 0.22 μm membrane filter. Subsequently, the samples were subjected to testing using high-performance liquid chromatography (HPLC) under the following chromatographic conditions: C18 reverse-phase column; column temperature maintained at 25 °C; flow rate set at 1 mL/min; mobile phase consisting of solvent A (acetonitrile) and solvent B (0.1% phosphoric acid gradient elution); detection wavelength set at 320 nm. The release of DQ from Aln-Lipo-DQ was studied using a dialysis method. Suitable concentrations of Lipo-DQ and Aln-Lipo-DQ samples were diluted to 3 mL and added to a dialysis bag (10 k Da MWCO). Then, 27 mL of pH 7.4 PBS (1% Tween80) was added to provide precipitation conditions. At scheduled time intervals, 1 mL of the external medium was sampled, and an equivalent volume of fresh medium was introduced to sustain sink conditions. The release of D or Q in the collected culture medium at each time point was analyzed using HPLC.

**Senescence-associated galactosidase (SA-β-Gal) staining**

BMMSCs were stained using the SA-β-Gal staining kit (Beyotime, Shanghai, China). The process involved washing the cells with PBS and subsequent fixation. The cells were then washed three times and incubated with freshly prepared SA-β-Gal staining solution in a CO₂ free incubator at 37 °C overnight. Optical images of the stained BMMSCs were captured using a microscope (Olympus, Japan). Senescent cells exhibited blue staining, while normal cells remained unstained. Three random fields of view were selected, and the numbers of senescent and total cells were manually counted.

**RT-qPCR analysis**

Total RNA was extracted from cells using a commercially available RNA isolation kit (Beyotime, China). The quantification and quality assessment of the RNA were then assessed using a NanoDrop One (Thermo Scientific™, USA). The cDNA Synthesis SuperMix (YEASEN, China) was then used to reverse transcribe RNA to cDNA. Subsequently, the qPCR reaction mixture was prepared with the synthesized cDNA, qPCR Master Mix (YEASEN), primers, and nuclease-free water. The mixture was then loaded into an Archimed real-time quantitative PCR system (ROCGENE, China) to perform the thermal cycling program. Data analysis was conducted using the 2^-ΔΔCt^ method, with Gapdh serving as the internal control for normalization. **Table S1** lists all primers used.

**Flow cytometry**

To identify surface markers on BMMSCs, cells were fixed and stained with various fluorescein-labeled antibodies, including CD44, CD29, Sca-1, CD34, and CD45 (Details on these antibodies are given in **Table S2**). The cells were then tested by flow cytometry using a CytoFLEX cytometer (Beckman Coulter, USA), and the resulting data were analyzed using FlowJo 10 software for analysis. For intracellular staining, including p16 and γ-H2AX, the cells were first fixed in Permeabilization and Fixation Solution. Subsequently, the cells were stained with an anti-γ-H2AX-PE antibody (diluted 1:100, Cell Signaling Technology, USA). Alternatively, they were stained with a primary rabbit anti-mouse p16 antibody (diluted 1:100, Abcam, USA). This was incubated with a goat anti-rabbit Alexa Fluor^®^ 647 secondary antibody, and the detection process was then initiated.

**Alizarin red staining**

The mineralization of cells following 21 days of osteogenic induction was assessed using Alizarin Red staining. The cells were first fixed and rinsed with PBS. Subsequently, they were stained with Alizarin Red S (Beyotime, Shanghai, China) for 5 min at room temperature. Calcium deposits were visualized using either photographic equipment or an inverted microscope (Life Technology, USA). For the purpose of quantitative analysis, the calcium nodules were first dissolved in a 10% solution of cetylpyridinium chloride to facilitate the release of calcium ions. The absorbance was measured at 562 nm using a microplate reader (BioTek, USA).

***In vitro* cytotoxicity assay**

The cytotoxicity of D and Q on BMMSCs was assessed using the Cell Counting Kit-8 (CCK-8) assay (Solarbio, China). BMMSCs were seeded at a density of 5×10^3^ cells per well in a 96-well plate and incubated overnight in a humidified incubator at 37°C with 5% CO_2_. Subsequently, the cells were treated with different concentrations of D and Q for 48 h. After the treatment, the medium was replaced with medium containing CCK-8 and incubated for 2 h, taking care to minimize bubble formation during the medium exchange to prevent any interference with the absorbance measurements. Finally, absorbance (OD) was measured at 450 nm using a microplate reader. Cell viability was calculated by comparing the average percentage of viable cells in the treatment groups to that of the untreated control group across six independent experiments.

**ALP staining**

Alkaline phosphatase (ALP) staining was performed using a commercially available ALP color development kit (Beyotime, Shanghai, China). BMMSCs were cultured in osteogenic induction medium for two weeks. According to the manual, cells were fixed and then stained with freshly prepared ALP staining solution. Regions with ALP activity reacted with the substrate in the staining solution, resulting in the formation of a blue product. The reaction was terminated with PBS, based on the staining intensity. The stained samples were observed and recorded under a microscope, with ALP-positive areas appearing blue. Quantitative analysis was performed using Image J software.

**Colony formation assay**

Bone marrow cells were obtained from femurs and tibias, and erythrocytes were lysed with RBC lysis buffer followed by neutralization with PBS. After centrifugation, the cells were resuspended in culture medium and prepared into single cell suspensions The cells were seeded at a density of 1 × 10^6^ cells per well in 60-mm dishes and incubated, with the culture medium changed every three days while monitoring colony formation. After two weeks, the cells were fixed and stained with crystal violet for 10 min, then carefully washed with water to remove excess dye, air dried, and quantitatively analyzed for the number of purple crystal violet-stained colonies using a stereomicroscope (Feica, Germany).

**Supplementary Tables S1 to S2**

**Table S1 Primers used in qPCR**

| **Gene** | **primer sequence (5’-3’)** |
| --- | --- |
| Gapdh | AGGTCGGTGTGAACGGATTTG |
|  | GGGGTCGTTGATGGCAACA |
| Mcp1 | CCACTCACCTGCTGCTACTCA |
|  | TGGTGATCCTCTTGTAGCTCTCC |
| Cxcl1 | GAACGCTGGCTTCTGACAAC |
|  | AATGTCCAAGGGAAGCGTCA |
| Il1β | ATCTCGCAGCAGCACATCAA |
|  | ATGGGAACGTCACACACCAG |
| Il6 | CTCTGGGAAATCGTGGAAAT |
|  | CCAGTTTGGTAGCATCCATC |

| **Antibody** | **Catalog** | **Manufacture** | **Dilution** |
| --- | --- | --- | --- |
| CD44 | 103012 | eBiolegend | 1:100 (FACS) |
| CD29 | 102205 | eBiolegend | 1:100 (FACS) |
| Sca-1 | 108111 | eBiolegend | 1:100 (FACS) |
| CD34 | 2414928 | eBioscience | 1:100 (FACS) |
| CD45 | 103114 | eBiolegend | 1:100 (FACS) |

**Table S2 Antibodies information**

**Supplementary Figs. S1 to S12**


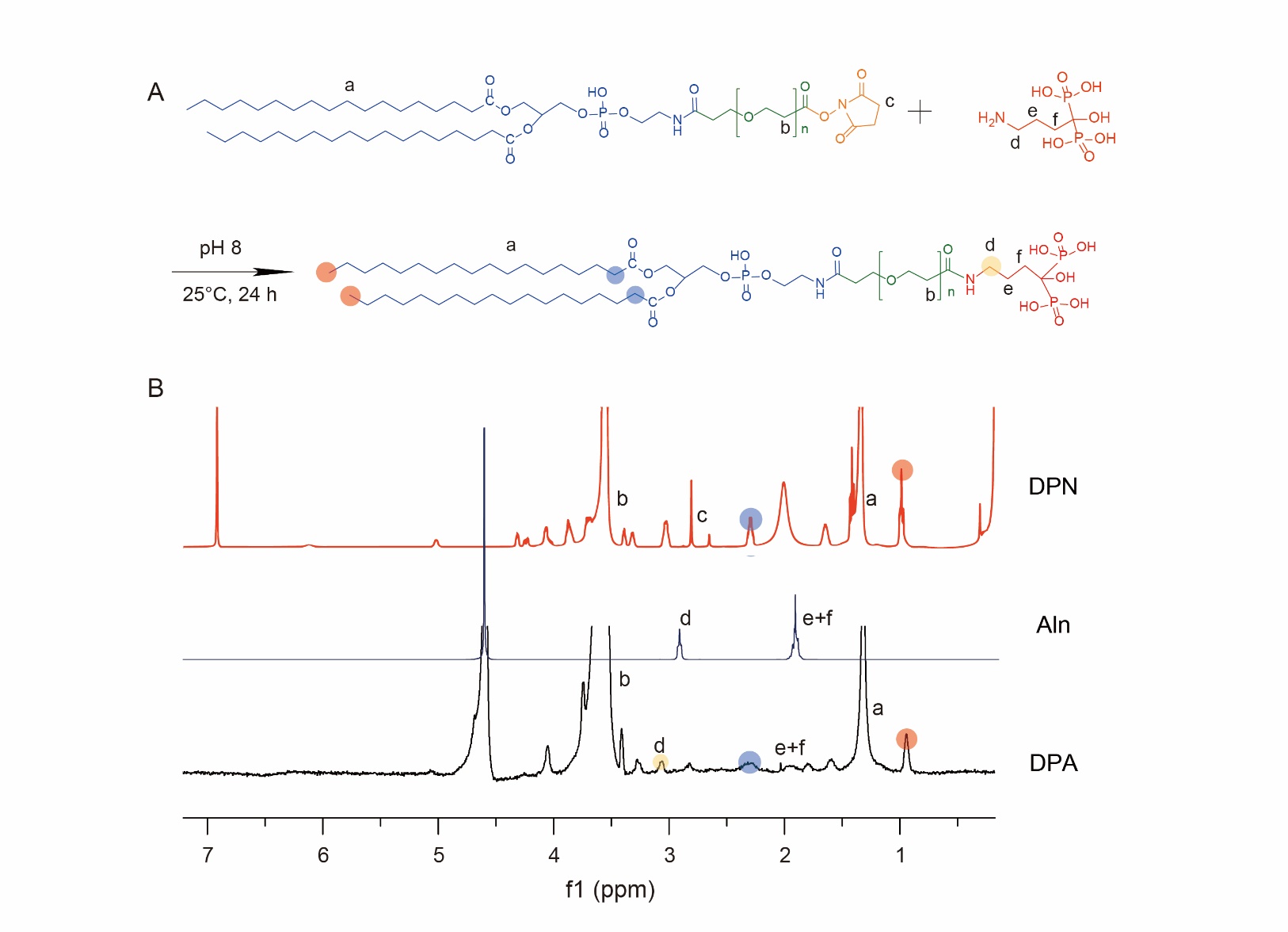


**Fig. S1**. **Synthesis and characterization of DSPE-PEG-Aln.** (A) Chemical synthesis of DSPE−PEG−Aln. (B) Chemical structure characterization by ^1^H NMR with DSPE-PEG-NHS (DPN) in DCl_3_ and alendronate (Aln), DSPE-PEG-Aln (DPA) in D_2_O confirmed the formation of DSPE-PEG-Aln conjugate.


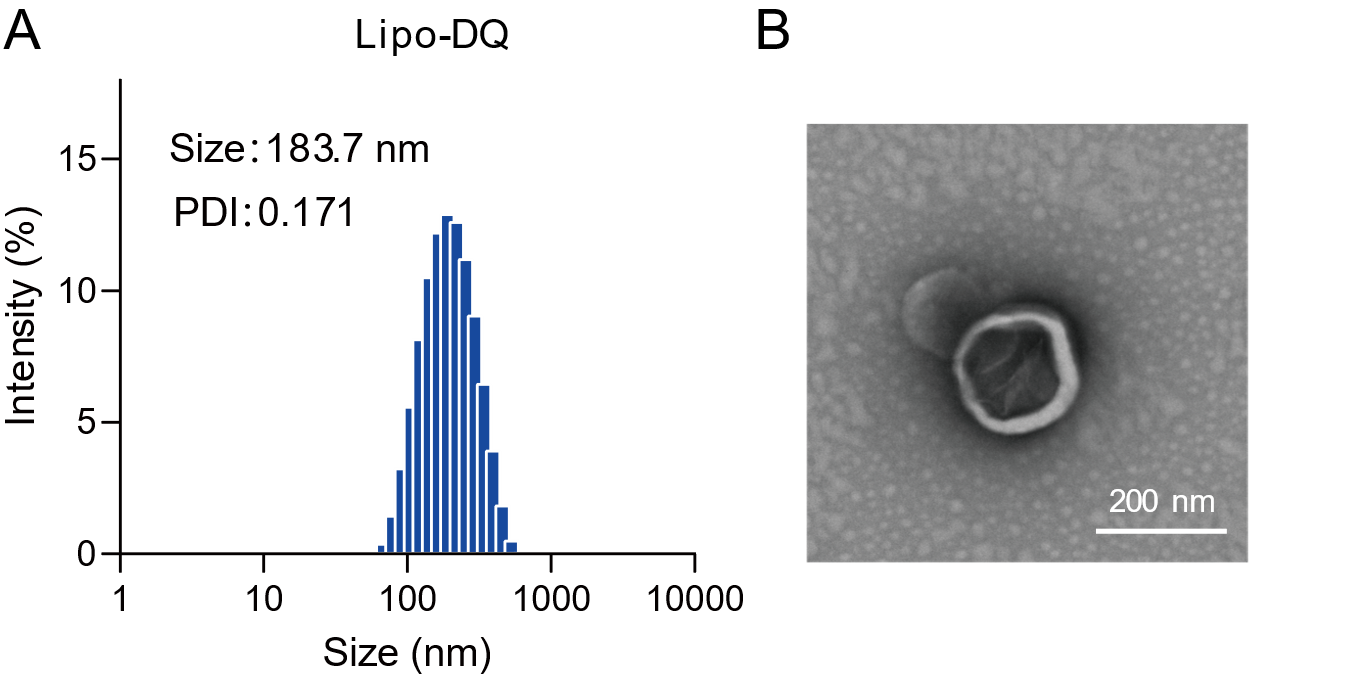


**Fig. S2**. **Characterization of Lipo-DQ.** (A) Particle size distribution of Lipo-DQ, and (B) a representative TEM image of Lipo-DQ (scale bar, 200 nm).


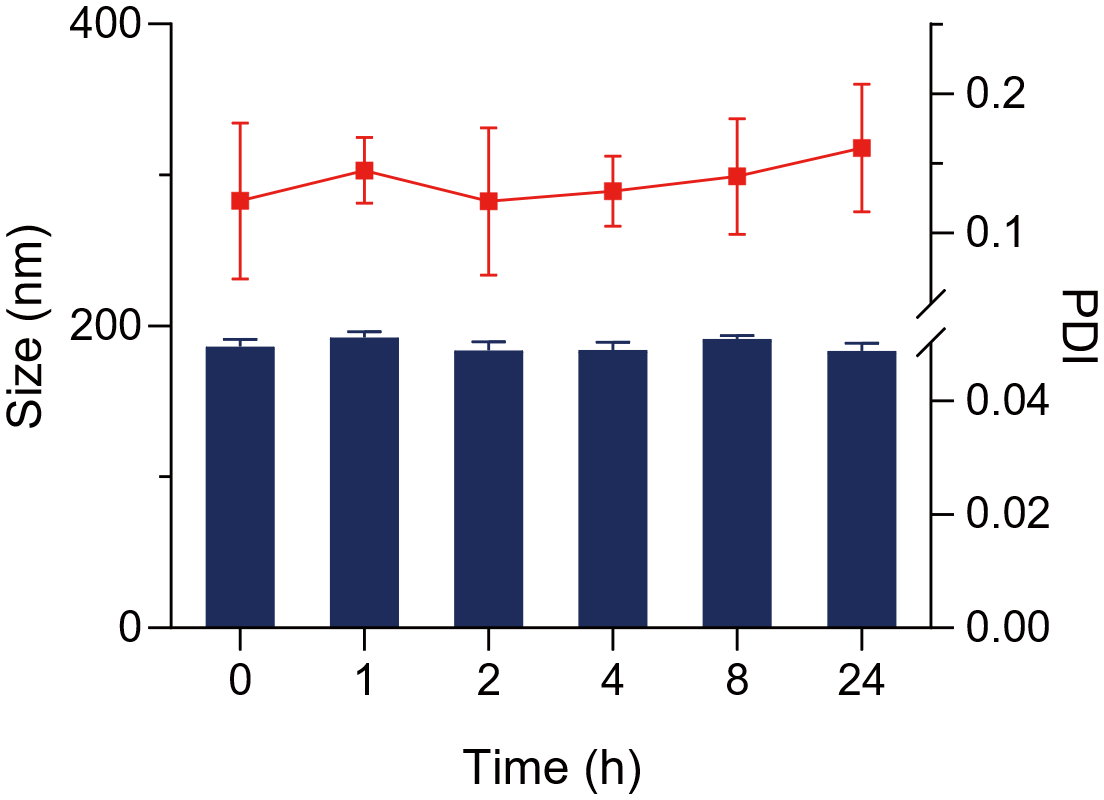


**Fig. S3. Serum stability of Aln-Lipo-DQ in fetal bovine serum within 24 h.** Particle size and PDI of Aln-Lipo-DQ were measured at 37°C in 10% fetal bovine serum. Measurements were taken at 0, 1, 2, 4, 8, and 24 h using DLS.

**Fig. S4**. **Aln-Lipo exhibits strong binding affinity to HAp.** The supernatant NIR images, along with their semi-quantification and precipitate images after incubation of different concentrations (0.1, 0.5, 1.0 mg/ml) of Aln-Lipo-Cy5.5 with HAp (n = 3). ***P* < 0.01 and *****P* < 0.0001.


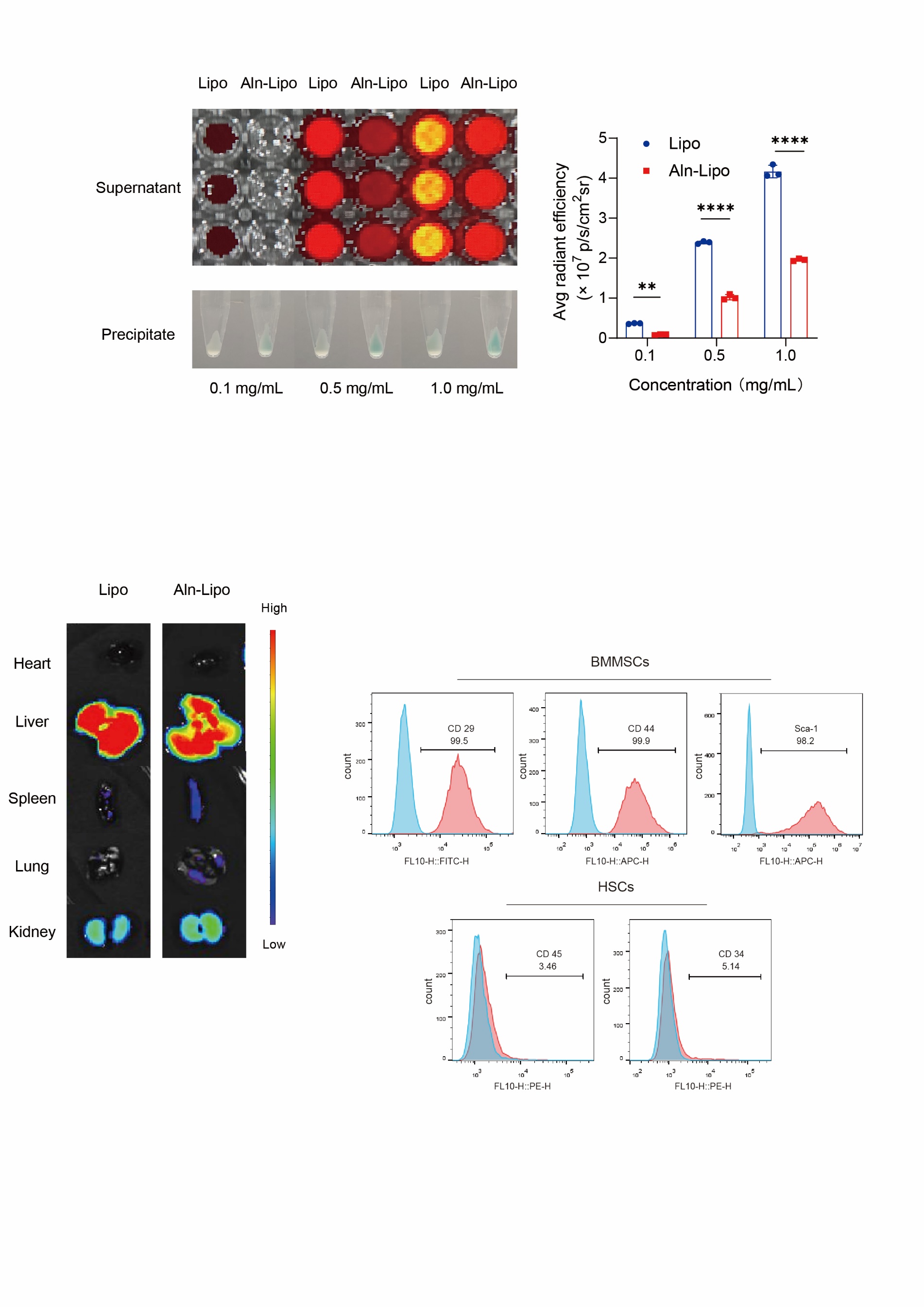


**Fig. S5**. **Identification of surface markers of BMMSCs.** Immunophenotypic analysis by flow cytometry showing cells expressing BMMSCs surface markers. Data are representative of cells from 3 mice.


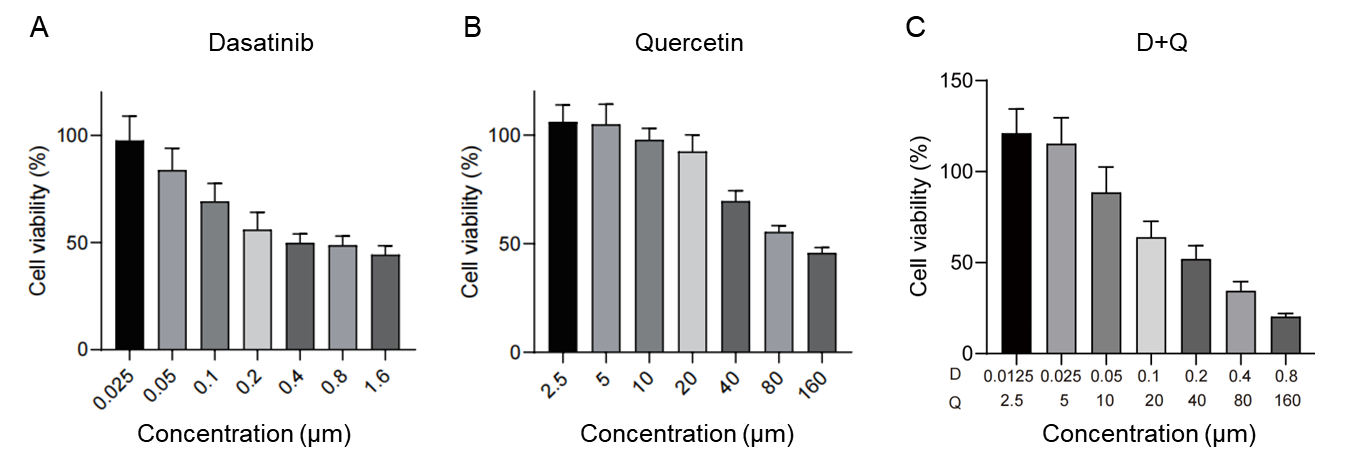


**Fig. S6. Cytotoxicity analysis of dasatinib and quercetin.** The toxic effects of (A) Dasatinib, (B) Quercetin, and (C) D + Q on normal BMMSCs were measured with CCK-8 kit. Data are presented as the mean ± SD.


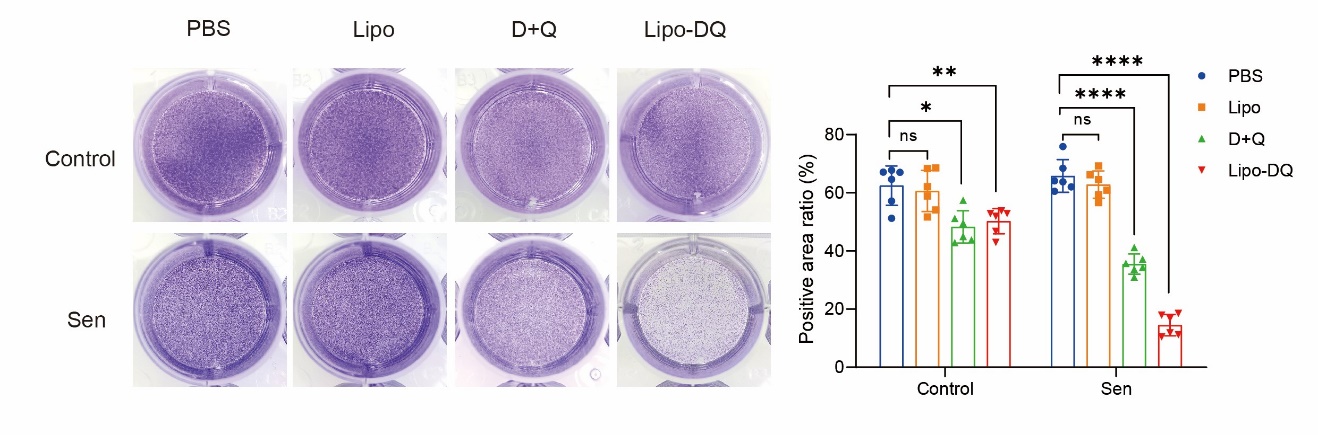


**Fig. S7**. **D + Q and Lipo-DQ exhibit strong cytotoxicity towards senescent BMMSCs, while having minimal effects on normal cells.** Normal and senescent BMMSCs were treated with D + Q, Lipo-DQ respectively for 48 h and then stained with crystal violet (n = 6). **P* < 0.05, ***P* < 0. 01 and *****P* < 0.0001.


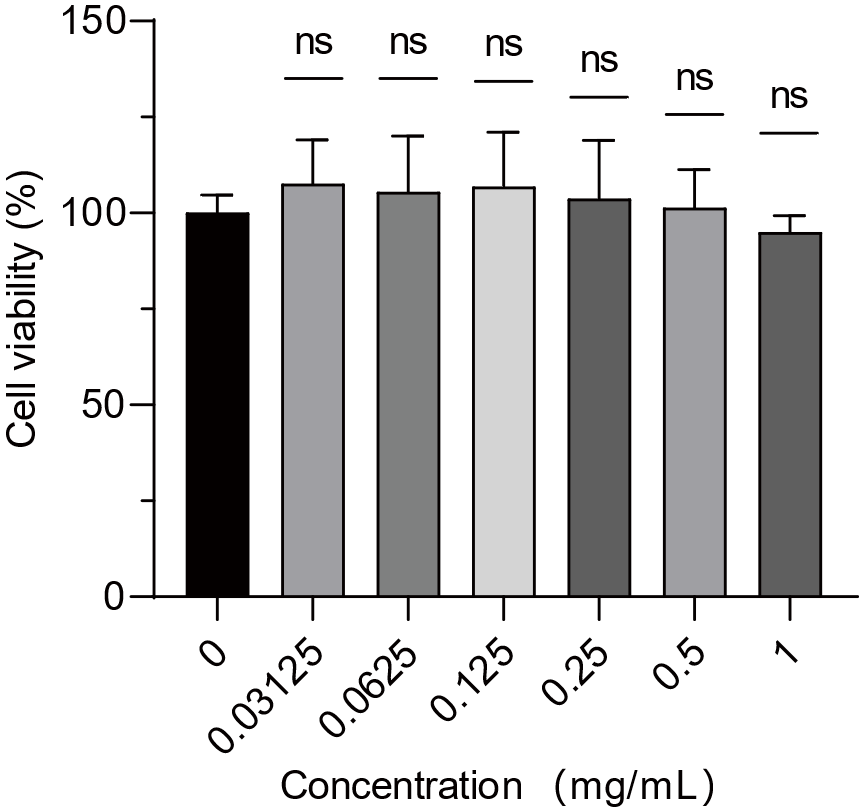


**Fig. S8. The *in vitro* cytotoxicity of Aln-Lipo.** The toxic effects of Aln-Lipo on normal BMMSCs were measured with CCK-8 kit. Data are presented as the mean ± SD.


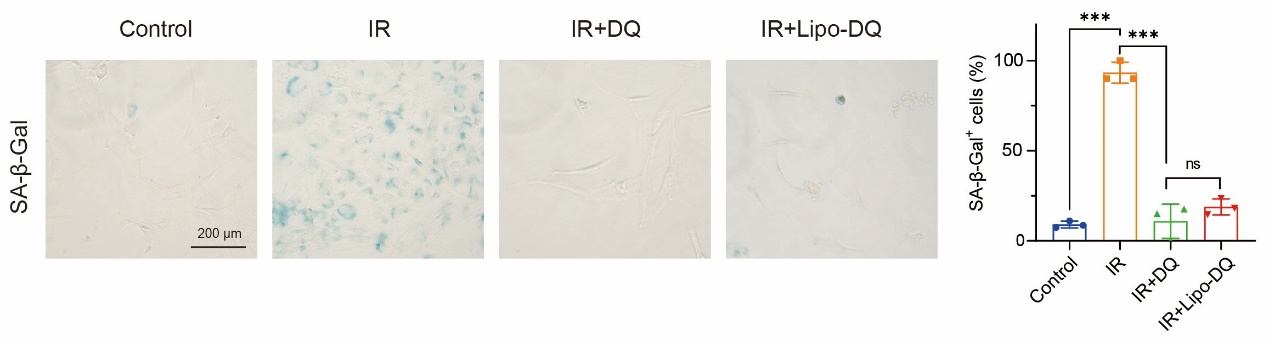


**Fig. S9**. **D + Q and Lipo-DQ reduce the proportion of SA-β-Gal^+^ cells in radiotherapy-induced senescent BMMSCs.** The percentage of SA-β-Gal^+^ cells was used to evaluate radiation-induced cellular senescence (n = 3). ****P* < 0.001.


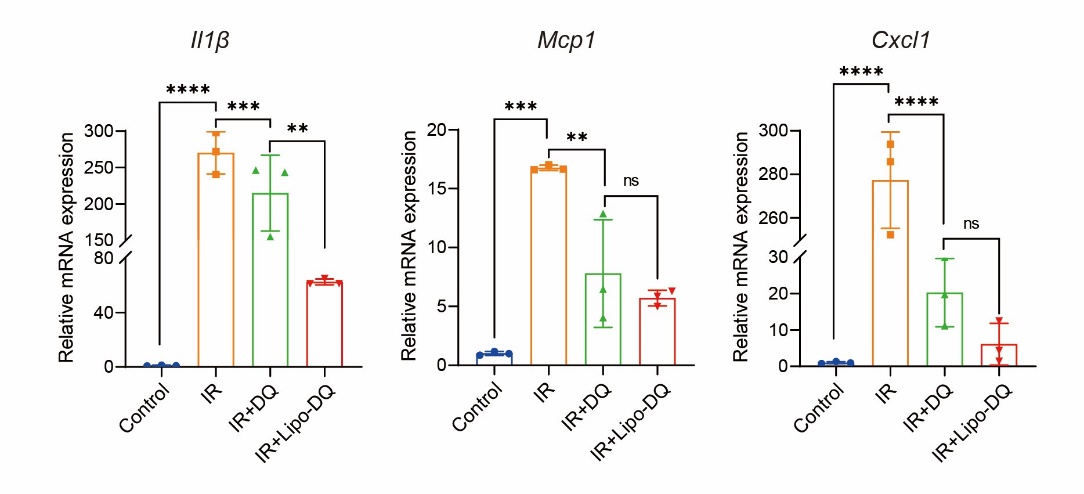


**Fig. S10**. **D + Q and Lipo-DQ reduce the mRNA expression levels of SASP in radiotherapy-induced senescent BMMSCs.** q-PCR analysis for the mRNA levels of *Ilβ, Cxcl1* and *Mcp1* in radiation-induced senescent BMMSCs treated with D + Q or Lipo-DQ (n = 3). ***P* < 0.01, ****P* < 0.001 and *****P* < 0.0001.


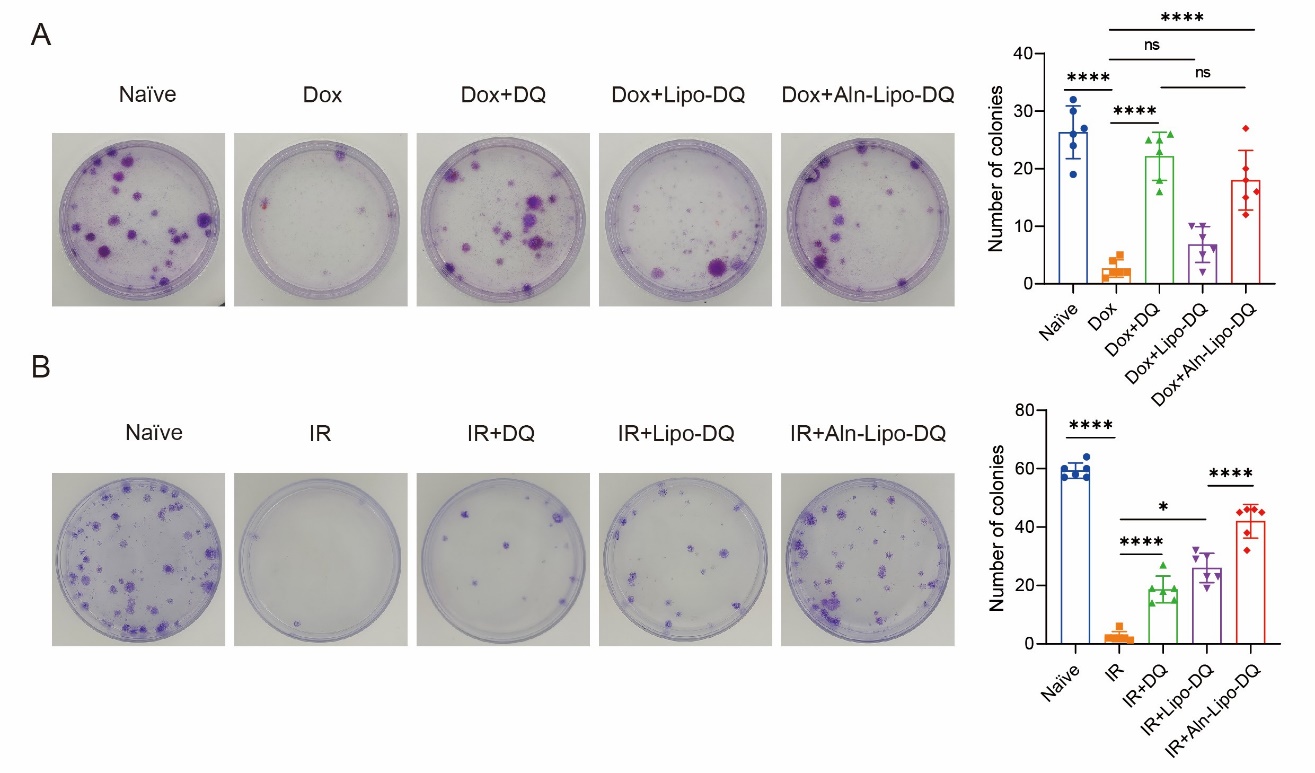


**Fig. S11**. **Colony formation assay of extracted BMMSCs from two osteoporosis mouse models treated with different treatment regimens.** Representative optical images of colony formation of different groups stained with crystal violet and quantification of the relative number of colonies are shown. (A) Dox-induced model, (B) radiotherapy-induced model **P* < 0.05 and *****P* < 0.0001.


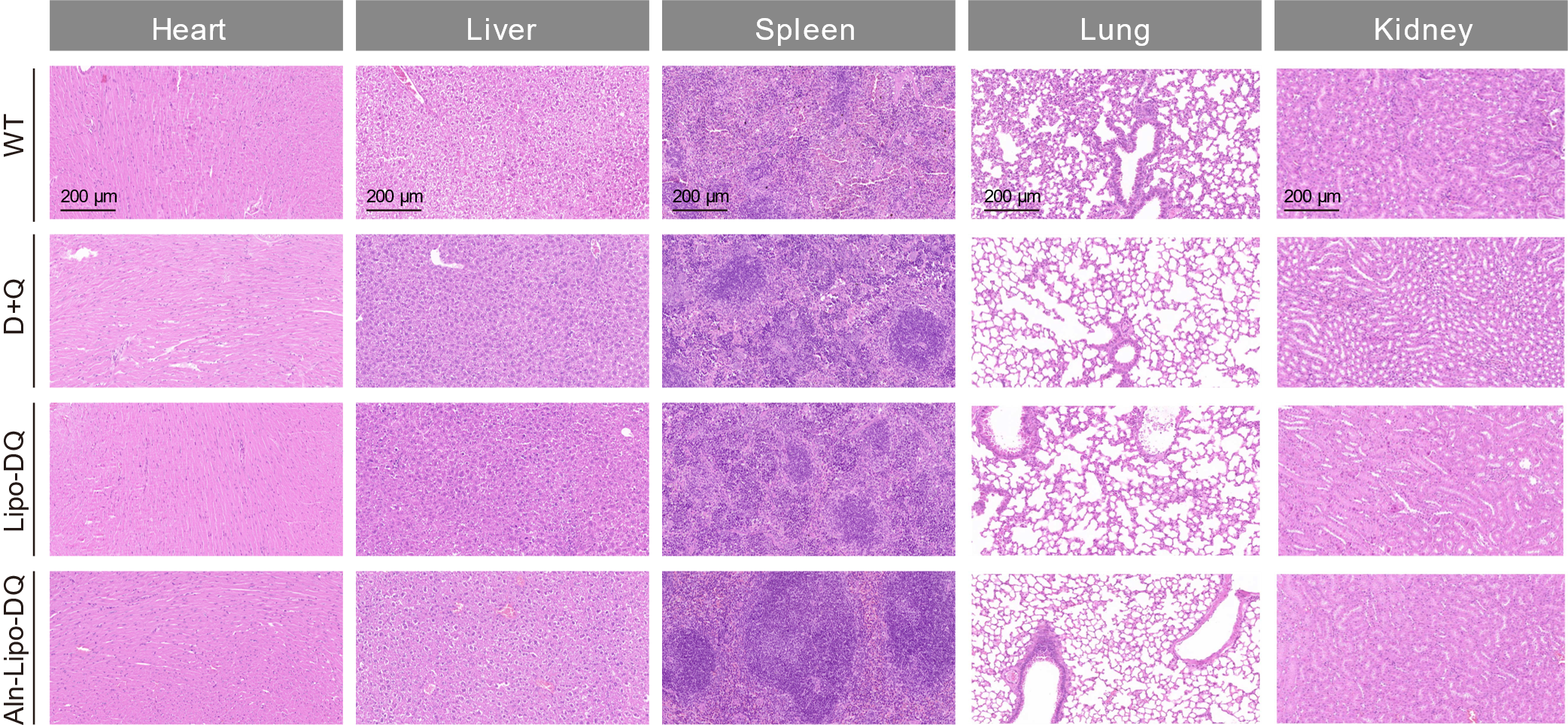


**Fig. S12. *In vivo* biosafety assessment of D + Q, Lipo-DQ, and Aln-Lipo-DQ.** Representative H&E staining images of the heart, liver, spleen, lungs, and kidneys from mice receiving various treatments. Scale bars are 200 μm.
